# Sustained Release of BMP-2 in Bioprinted Alginate for Osteogenicity in Mice and Rats

**DOI:** 10.1371/journal.pone.0072610

**Published:** 2013-08-19

**Authors:** Michelle T. Poldervaart, Huanan Wang, Johan van der Stok, Harrie Weinans, Sander C. G. Leeuwenburgh, F. Cumhur Öner, Wouter J. A. Dhert, Jacqueline Alblas

**Affiliations:** 1 Department of Orthopaedics, University Medical Center Utrecht, Utrecht, The Netherlands; 2 Department of Biomaterials, Radboud University Nijmegen Medical Center, Nijmegen, The Netherlands; 3 Department of Orthopaedics, Erasmus University Medical Center Rotterdam, Rotterdam, The Netherlands; 4 Department of Biomechanical Engineering, Delft University of Technology, Delft, The Netherlands; 5 Department of Rheumatology, University Medical Center Utrecht, Utrecht, The Netherlands; 6 Faculty of Veterinary Medicine, Utrecht University, Utrecht, The Netherlands; National University of Ireland, Galway, Ireland

## Abstract

The design of bioactive three-dimensional (3D) scaffolds is a major focus in bone tissue engineering. Incorporation of growth factors into bioprinted scaffolds offers many new possibilities regarding both biological and architectural properties of the scaffolds. This study investigates whether the sustained release of bone morphogenetic protein 2 (BMP-2) influences osteogenicity of tissue engineered bioprinted constructs. BMP-2 loaded on gelatin microparticles (GMPs) was used as a sustained release system, which was dispersed in hydrogel-based constructs and compared to direct inclusion of BMP-2 in alginate or control GMPs. The constructs were supplemented with goat multipotent stromal cells (gMSCs) and biphasic calcium phosphate to study osteogenic differentiation and bone formation respectively. BMP-2 release kinetics and bioactivity showed continuous release for three weeks coinciding with osteogenicity. Osteogenic differentiation and bone formation of bioprinted GMP containing constructs were investigated after subcutaneous implantation in mice or rats. BMP-2 significantly increased bone formation, which was not influenced by the release timing. We showed that 3D printing of controlled release particles is feasible and that the released BMP-2 directs osteogenic differentiation *in vitro* and *in vivo*.

## Introduction

For the treatment of large bone defects currently auto- or allograft bone grafts are being used. Both graft types have drawbacks such as limited availability of donor tissue, donor site morbidity (autograft) or inferior performance (allograft) [1], [2]. Tissue engineered bone constructs could ideally reduce the need for donor bone; this is an important pillar in the field of regenerative medicine. Within these constructs signaling molecules such as growth factors are used to induce cellular growth, proliferation and differentiation [3], [4].

BMP-2 is a potent osteoinductive growth factor (both *in vitro* and *in vivo*) that belongs to the transforming growth factor-β (TGF-β) protein superfamily. BMP-2 stimulates MSCs towards osteogenic differentiation [5] and has been used in the clinic for spinal fusion surgery and tibial fracture healing [6]. Protein delivery via a collagen sponge results in a BMP-2 presence of up to 8 days locally [7], resulting from a half-life of only 7–16 minutes systemically, due to fast degradation by proteinases [8]. Studies have shown that BMP-2 containing products that are used in the clinic release the protein with a large initial burst [9]. The dosage used in these clinical applications is much higher than the effective dose in order to compensate for the fast wash-out of the protein. The use of BMP-2 in spinal fusion is associated with an increased risk of bone overgrowth, problems due to exposed dura/nerves and osteolysis. An increased risk of malignancies is suspected for products applying the highest concentration [6], [10]. In uncomplicated fracture healing, the gene coding for BMP-2 is upregulated for about four weeks after fracture, which supports the approach to strive for a more sustained release of the BMP-2 protein at lower dosage, as an alternative and effective strategy in enhancing osteogenic differentiation and bone formation while reducing the risk of side effects and complications [11]–[13]. To accomplish such a gradual and sustained release many controlled release vehicles have been described in literature; gelatin microparticles (GMPs) are particularly promising because growth factors can be incorporated into these microspheres by simple post-loading, thereby avoiding chemical reactions that can damage the activity of fragile proteins such as BMP-2. The post-loading strategy is facilitated by the formation of polyion complexes between charged amino acid residues present in both growth factors and gelatin macromolecules. In addition, GMPs are non-cytotoxic, biodegradable and have already been used in numerous formulations as isolated particles or incorporated as dispersed phase into hydrogels to deliver BMP-2 and other growth factors such as TGF-β1 and bFGF [14], [15].

The performance of hydrogels as scaffold materials can be improved by introducing porosity, which lowers the diffusion distance of oxygen and other nutrients to the center of the construct [16]. By applying the bioprinting technology, porosity of hydrogel-based constructs is easily achieved. In addition, the technique of 3D fiber deposition enables production of scaffolds with a defined architecture and regional differences by computer controlled deposition of the cell- and growth factor laden hydrogel [17]. A well-known hydrogel is alginate, an non-toxic biocompatible anionic polysaccharide polymer, which gelates by addition of divalent cations such as Ca^2+^, forming reversible ionic bridges between the polymer chains [18]. Alginate is very suitable as a material for 3D printing because it is a viscous, cell-friendly and rapidly crosslinkable hydrogel in which bone formation has been previously reported [19]–[21].

The present study combined controlled growth factor release with bioprinting technology to enable production of hydrogel scaffolds with properties that can be tuned both in time and space. This study describes bioprinting of hydrogel scaffolds in which controlled release particles containing BMP-2 are included. We investigated whether prolonged BMP-2 presence in scaffolds promotes osteogenic differentiation and bone formation compared to fast growth factor release. Osteogenic differentiation and bone formation in the composite hydrogel scaffolds were observed *in vitro* and *in vivo,* respectively.

## Materials and Methods

### Experimental design

Three experimental groups are investigated in consecutive experimental settings throughout the paper, namely: 1. Control group with empty gelatin microparticles (GMPs) (i.e. PBS-laden), 2. Fast release group with empty GMPs and BMP-2 added directly to the hydrogel, 3. Slow release group with BMP-2-loaded GMPs, dispersed in the hydrogel (Table 1). BMP-2 concentrations were adjusted to the calculated effective dose per experimental setup, based on literature [22]–[24].

**Table 1:** Experimental groups.

Model	Group	GMPs	[BMP-2]	Method	Hydrogel	Ceramic	Cells
*In vitro*	1. Control	PBS laden	None	Bioprinted	Alginate	None	gMSCs
	2. Fast	PBS laden	25 ng/ml				
	3. Slow	BMP-2 laden	25 ng/ml				
Mouse	1. Control	PBS laden	None	Bioprinted	Alginate	Granules	gMSCs
	2. Fast	PBS laden	250 ng/ml			106–212 µm	
	3. Slow	BMP-2 laden	250 ng/ml				
Rat	1. Control	PBS laden	None	Cast	Matrigel	Cylinder	None
	2. Fast	PBS laden	100 µg/ml			7 mm ∅	
	3. Slow	BMP-2 laden	100 µg/ml			3 mm height	

*In vitro* we investigated release kinetics and bioactivity of BMP-2 from GMPs. Osteogenic differentiation was monitored in bioprinted, cell-laden alginate constructs *in vitro* and *in vivo*, after subcutaneous implantation in mice, using flow cytometry and immunohistochemistry. All investigative groups were subsequently combined with ceramic cylinders and implanted subcutaneously in rats to study bone formation using histomorphometry.

### Production of gelatin microparticles (GMPs)

We adapted the protocol previously described by Tabata et al. [25], [26]. A water in oil emulsion was made using water dissolved gelatin type B (Sigma, St Louis, MO, USA) 10% (w/v) that was added drop wise to refined olive oil (Arcos Organics, NJ, USA) at 60° Celsius. The solution was stirred (350 rpm) for 15 minutes and then rapidly cooled with an ice bath to induce gelation of gelatin droplets. The microparticles were washed with 150 ml of chilled acetone and filtered under pressure (filter paper grade 2, Whatman, Tokyo, Japan). The microparticles were sieved and covalently cross-linked overnight using a 10.6 mM aqueous glutaraldehyde solution (Merck, Darmstadt, Germany) [27], [28]. Three washing steps with 100 mM glycine solution (Sigma, St Louis, MO, USA) were applied to remove residual aldehyde groups. Subsequently, the microparticles were washed in deionized water (MilliQ) three times, freeze-dried overnight and kept at 4°C in a vacuum container until use. The microparticles were loaded with the dissolved growth factor by diffusional loading.

### Radiolabeling of BMP-2

Human recombinant BMP-2 (InductOS, Wyeth/Pfizer, New York, NY, USA) was labeled with ^125^I according to the iodogen method [29]. Seventy-five µl of a 0.337 mg/ml BMP-2 solution was pipetted into a 100 µg iodogen coated tube containing 10 µl ^125^I (radioactivity = 1000 mCi, Perkin-Elmer, Boston, MA, USA). A 0.5 M phosphate buffer solution with a pH of 7.0 was added to a total volume of 100 µl. This was incubated for 10 minutes at room temperature, then 100 µl of saturated tyrosine solution in PBS was added to react with unbound ^125^I in the solution. The reaction mixture was filtered over Sephadex G25M column (PD-10, Pharmacia, Uppsala, Sweden) and eluted with 1 mM NaCl with 0.5% BSA (pH 7.0). The fraction with the highest radioactivity was used for the release study. The resulting ^125^I-BMP-2 solution was diluted to a concentration of 12 µg/ml for further use.

### In vitro release of BMP-2 measured by radiolabeling

Microspheres were loaded with ^125^I-BMP-2 by diffusional loading. 25 µl of a 12 µg/ml ^125^I-BMP-2 solution was placed on 5 mg microspheres (n = 4) and kept at 4° Celsius overnight to allow complete growth factor absorption. These samples, each containing 300 ng ^125^I-BMP-2, were put in PBS with 400 ng/ml bacterial collagenase type 1A (Sigma, St Louis, MO, USA) and 0.001 % (w/v) sodium azide (Wako, Kyoto, Japan) as the release medium. The release medium was refreshed at multiple time points after centrifuging for 5 minutes at 10,000 rpm to spin down the particles. Release was determined by measuring the residual γ-irradiation in the supernatant using a shielded γ-counter (Wizard, Pharmacia-LKB, Uppsala, Sweden) and corrected for radioactive decay.

### In vitro release of BMP-2 measured by ELISA

Microspheres (25 mg) were loaded with 10 µg/ml BMP-2 in PBS/0.5 % BSA by diffusional loading overnight at 4°C. Samples were placed into the top compartments of a Transwell system (0.4 µm pores, Corning Sigma, St Louis, MO, USA). The release medium (PBS/0.5 % BSA) in the lower compartment was refreshed at multiple time points. Release was determined by measuring the amount of BMP-2 in the release medium samples in duplicate by ELISA (R&D, Minneapolis, MN, USA). Data are expressed as cumulative release in % of total input.

### Cell culture

gMSCs were obtained from bone marrow aspiration from iliac wings of adult Dutch milk goats (n = 3). This procedure was performed with permission of the local Ethical Committee for Animal Experimentation in compliance with the Institutional Guidelines on the use of laboratory animals. MSCs were isolated by their adherence to plastic tissue culture flasks. They were cultured in expansion medium, consisting of alpha minimum essential medium (α-MEM, Gibco, Breda, The Netherlands) that was supplemented with 15 % (v/v) fetal calf serum (Cambrex), 100 U/ml penicillin with 100 µg/ml streptomycin (Gibco), and 2 mM L-glutamine (Glutamax, Gibco). All cells were cultured in a humidified incubator at 37°C and 5 % CO_2_.

### Hydrogel preparation

Alginate powder (IMCD, Amersfoort, the Netherlands) was sterilized by UV-radiation and dissolved at a concentration of 30 mg/ml in α-MEM (Gibco) supplemented with 25 mM CaCl_2_. This prepolymerised alginate has a suitable viscosity for 3D-printing. gMSCs were added to the gel in a concentration of 10^7^ cells/ml, and for the *in vivo* scaffolds 5% (w/v) biphasic calcium phosphate granules (BCP-1150 containing 82 % HA and 18 %TCP, Xpand, Bilhoven, the Netherlands) with a diameter of 106–212 µm were added to improve scaffold osteoconductivity. Matrigel (growth factor reduced, BD, New Jersey, USA) was thawed overnight at 4 °C.

### Bioprinting

**Figure 1:**
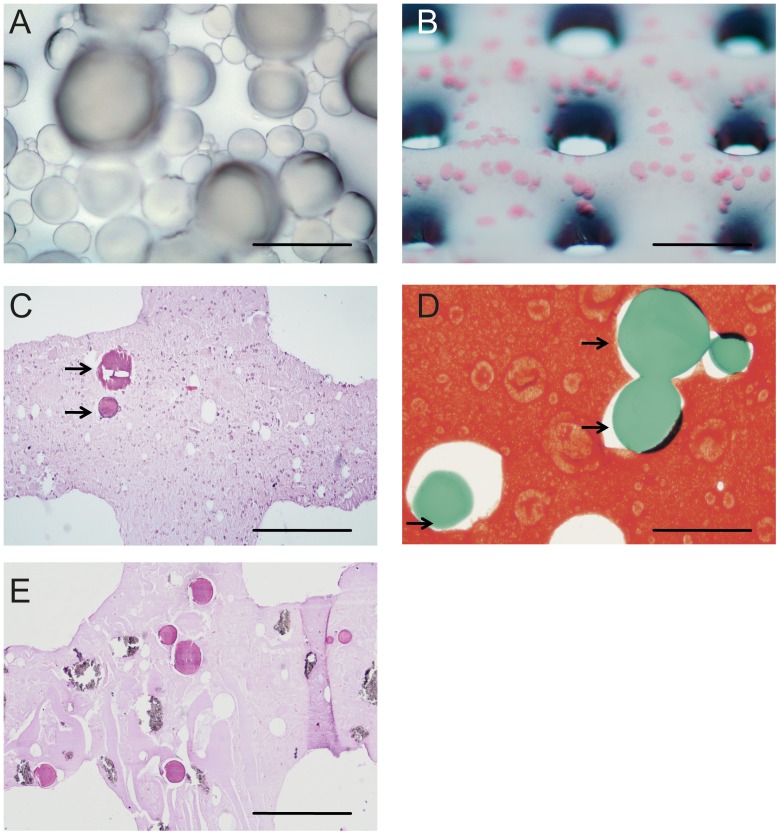
Bioprinted gelatin microparticles. A. Gelatin microparticles; B. GMPs in alginate, directly after printing, GMPs in pink; C. Section of bioprinted alginate construct, directly after printing (H/E staining). Arrows indicate GMPs; D. Safranin O staining of bioprinted alginate construct, directly after printing. Arrows indicate GMPs; E. Section of bioprinted alginate construct containing BCP, after 3 weeks of culturing (H/E staining). Experiments were performed in triplicate, representative pictures are shown. Scale bars indicate in A: 200 µm, B: 2 mm, C,E: 500 µm, D: 100 µm.

The Bioscaffolder pneumatic dispensing systems (SYS+ENG, Gladbeck, Germany) was used for bioprinting of the hydrogel scaffolds [17]. Blocks of 20×20×3 mm with vertically connected pores were designed with CAD/CAM software and translated to the NC-code for layer by layer material deposition (Figure 1). Scaffolds were printed in a laminar flow-cabinet into tissue culture plates. After printing, the scaffolds were crosslinked by adding 100 mM CaCl_2_ dissolved in MilliQ. After 15 minutes, the CaCl_2_ solution was removed, the constructs washed with Tris buffered saline (TBS) and cultured in 5 ml culture medium. Directly after printing one sample of each group was processed for paraffin embedding and stained with hematoxylin and eosin to assess the distribution of the different components within the constructs. 

### In vitro analysis of osteogenic differentiation

Three groups of constructs with a volume of 600 µl were bioprinted (alginate 3% and 10^7^ cells/ml in medium): 1. Control with empty GMPs (PBS laden), 2. Fast release with empty GMPs (PBS laden) and 25 ng/ml BMP-2 in the alginate, 3. Slow release with 25 ng/ml BMP-2 loaded on 2 mg GMPs. BCP granules were omitted from these constructs to allow cell retrieval for FACS analysis (Table 1). Constructs (n = 3) were cultured for three weeks in expansion medium in a humidified incubator at 37°C and 5% CO_2_. Part of the constructs was paraffin-embedded for histological evaluation, the rest immersed in citrate buffer (150 mM NaCl, 55 mM sodium citrate and 20 mM EDTA in H_2_O) for 15 minutes at 37°C to dissolve the alginate. The cell pellet was washed in PBS/BSA 5% (w/v) three times and stained for flow cytometry. This process was repeated with the gMSCs of three different donors.

### ALP analysis by FACS staining

Retrieved cells of each group (n = 3) were incubated with the monoclonal anti-human alkaline phosphatase (ALP) conjugated with Alexa fluor 647 (clone B4-78, BD Pharmingen, NJ, USA) diluted 1∶100 in PBS/1 % (v/v) FCS (Cambrex) in the dark for an hour at 4°C. Control stainings were performed using isotype-matched control antibody (IgG_1_). After antibody incubation cells were washed and taken up in PBS/FCS and analyzed by FACS Calibur.

Data were analyzed using a one-way ANOVA with Bonferroni correction and expressed as mean±SD. A value of p<0.05 was considered statistically significant.

### Bioprinted constructs for in vivo analysis of osteogenic differentiation

Three groups of constructs (Table 1) with a volume of 600 µl were bioprinted (20×20×3 mm, alginate 3% and 10^7^ cells/ml in medium) as follows: 1. Control with empty GMPs (PBS laden), 2. Fast release with empty GMPs (PBS laden) and 250 ng/ml BMP-2 in the alginate, 3. Slow release with 250 ng/ml BMP-2 loaded on 2 mg GMPs. Constructs were trimmed to a size of 10×5×3 mm and kept in expansion medium overnight before implantation.

### BCP cylinders for bone formation analysis

Biphasic calcium phosphate cylinders (BCP-1150 containing 82 % HA and 18 %TCP, Xpand, Bilhoven, the Netherlands) 7 mm in diameter, 3 mm in height with a porosity of 75±1 % were filled with 100 µl Matrigel, supplemented as follows (Table 1): 1. Control with empty GMPs (PBS laden), 2. Fast release with empty GMPs (PBS laden) and 100 µg/ml BMP-2 in 100 µl Matrigel, 3. Slow release with 100 µg/ml BMP-2 loaded on 1 mg GMPs in 100 µl Matrigel.

### Ethics Statement

This study was carried out following the Institutional Guidelines under the Dutch Law (“Wet op de dierproeven”) on the use of laboratory animals, in accordance with the recommendations in the Guide for the Care and Use of Laboratory Animals of the National Institutes of Health. The study protocol was approved by the Dutch Ethical Committee for Animal Experimentation (Dier Experimentele Commissie, DEC) of the University of Utrecht, the Netherlands (Permit Number: 06/248). All surgery was performed under isoflurane anesthesia, and all efforts were made to minimize suffering.

### In vivo implantation

Six female nude mice (Hsd-cbp NMRI-nu, Harlan, Boxmeer, The Netherlands), 6 weeks of age and 10 male Wistar rats (Charles River), 16 weeks of age were housed in standard cages at the Central Laboratory Animal Institute. They were allowed to acclimatize at the institute for at least two weeks prior to surgery. Surgery was performed under inhalation anesthesia of 3 % isoflurane. After skin incision subcutaneous pockets were made by blunt dissection. The 3D printed scaffolds were placed in one of the four dorsal pockets in the mice. The BCP cylinders were placed subcutaneously in one of the six dorsal pockets in the rats. All scaffolds were assigned to a subcutaneous pocket by randomization. Pockets were closed using sutures (Vicryl 4.0). Postoperatively the animals were weighed and given a subcutaneous injection of buprenorphin (0.05 mg/kg, Temgesic, Schering-Plough/Merck, Whitehouse station, NJ, USA) every 8 hours (3 times in total).

### Sample processing

The mice were terminated by cervical dislocation 6 weeks after implantation. The scaffolds were retrieved to analyze osteogenic differentiation. Samples were fixed overnight in 4% buffered formalin supplemented with 100 mM CaCl_2_ and further dehydrated for paraffin embedding. The rats were terminated with an injection of Euthanasol. Scaffolds were retrieved and scanned with micro-CT. Subsequently they were fixed in 4% buffered formalin, dehydrated using ethanol series and embedded in polymethylmethacrylate (MMA).

### Histology

Hematoxylin/eosin (HE) staining was performed on all paraffin embedded samples to investigate the properties of the 3D printed scaffolds directly after printing, after *in vitro* culturing for 3 weeks and after 6 weeks of *in vivo* implantation.

Safranin O staining was performed to investigate GMP distribution throughout the scaffold directly after printing and to monitor alginate degradation after *in vivo* implantation. Sections were incubated for 5 minutes with Weigert's hematoxylin after deparaffinisation, washed for 5 minutes in running tap water, rinsed in distilled water and counterstained with 5.2 mM Fast Green solution. Sections were then rapidly rinsed 3 times in 166.5 mM acetic acid (Merck) and counterstained again with a freshly prepared 3.56 mM Safranin O staining (Merck). Both cultured and mouse implanted samples were immunostained for osteocalcin, a marker for osteogenic differentiation. The cultured samples were incubated with a mouse-anti-osteocalcin primary antibody (TaKaRa M044, clone OCG4) at 40 µg/ml and a goat-anti-mouse-IgG-HRP (Dako P0447) secondary antibody at 5 µg/ml. The implanted samples were incubated with rabbit-anti-osteocalcin primary antibody (Enzo ALX-210-333-C100) at 4 µg/ml and a goat-anti-rabbit IgG-HRP (Dako P0448) secondary antibody at 3.3 µg/ml. All antibodies were diluted in TBS containing 5% BSA and 100 mM CaCl_2_, wash buffer consisted of TBS supplemented with 0.1% (v/v) Tween20 (TBST). Stainings were developed with diaminobenzidine (DAB) and Mayer's hematoxylin was used for counterstaining.

Sections of the bioprinted *in vivo* samples were deparaffinised and incubated with Weigert's hematoxylin, followed by Goldner's trichrome to assess collagen deposition. The MMA embedded samples were sawed centrally into 25 µm thick sections using a sawing microtome (Leica, Nussloch, Germany) and were stained with methylene blue and basic fuchsin for histomorphometric analysis. High-resolution digital pictures of the samples were taken using transmitted light microscopy (Olympus-BX50, Olympus, Zoeterwoude, the Netherlands). Bone and scaffold were pseudo-colored red and green using Adobe Photoshop CS5.1 and respective surfaces were measured (Adobe Systems Inc, San Jose, USA). Bone area percentage was calculated as (bone area/(total area - BCP area)*100%.

### Micro-CT evaluation

Immediately after explantation of the subcutaneously implanted BCP scaffolds, *ex vivo* micro-CT scans were acquired using a 9 µm-resolution protocol (65 kV, 1320 ms exposure time, 1.0 mm Al filter, 0.32 degree rotation step, 53 min scan). All micro-CT images were reconstructed using volumetric reconstruction software NRecon version 1.5 (Bruker micro-CT). The BCP scaffold material was excluded through segmentation of the greyscale images with a global threshold. Within the remaining pore space, a second global threshold was applied to segment out calcified tissue from non-calcified tissue and noise. Subsequently bone was measured after an extra erosion step was applied to reduce border artifacts caused by the BCP scaffold. All analysis and segmentation was performed by a blinded observer.

### Statistics

Histomorphometry data were analyzed by SPSS version 20 software (IBM, Chicago, Illinois, USA). Differences between groups were analyzed with a paired t-Test. *Post hoc* testing was performed using a Bonferroni correction. Significance was assumed when p<0.05.

## Results

### Gelatin microparticles

Particles were uniform in shape when analyzed using light microscopy (Figure 1A). GMPs were selected in the size range between 75–125 µm out of three size ranges (0–50 µm/50–75 µm/75–125 µm) since preliminary tests revealed that BMP-2 release from these spheres lasted up to three weeks (data not shown). Smaller particles exhibited a faster growth factor release, whereas release from larger particles was not tested since they could hinder 3D printing with smaller gauged syringes [30].

### In vitro release of BMP-2

**Figure 2:**
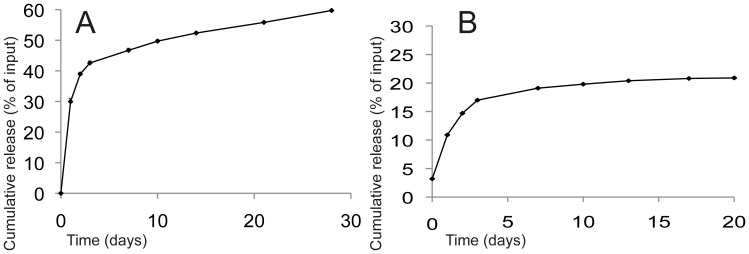
Cumulative release profiles of BMP-2 from gelatin microspheres. A. Detection of ^125^I-BMP-2 in PBS supplemented with collagenase, performed in triplicate. Results are presented as mean±SD (small SDs hardly visible in figure); B. BMP-2 concentrations in PBS without collagenase, as determined by ELISA.

To investigate whether GMPs could prolong release of BMP-2, several release experiments were performed. After overnight diffusional loading of BMP-2 solution, the amount of BMP-2 that was present in the release medium was measured at multiple time points up to 28 days. In figure 2A the cumulative release profile of loading the microspheres with an ^125^I-BMP-2 solution in PBS with collagenase A is shown, based on ^125^I signal in the medium. The reproducibility of the experiment was very high, standard deviations are less than 3 % and thus hardly visible in the figure. In figure 2B the release of BMP-2 into PBS without collagenase presence is shown, as measured with ELISA. An initial burst release of about 20–30% of the BMP-2 was observed during the first day, which was followed by a more sustained release for the following four weeks. Without collagenase (Fig. 2B), the initial burst of around 10 % occurred in the first day, followed by a slow release until day 10 whereafter the growth factor was not further released from the microspheres.

### Bioprinting of controlled release particles

The GMPs were embedded in the alginate hydrogel and 3D printed scaffolds were produced. Upon preparation of the 3D printed scaffolds, GMPs were evenly distributed throughout the construct (Figure 1B). Constructs remained intact for at least three weeks *in vitro*. Subsequently, we printed porous constructs containing GMPs (without BMP-2/fast release/slow release) and gMSCs from 3 different donor goats. One construct from each donor and group was embedded into paraffin directly after printing and stained with HE for gross histological evaluation. We observed that scaffold architecture was well preserved. Note the crossing of two hydrogel strands in the center of the picture (Figure 1C). Two GMPs are in focus (arrows), others were lost during tissue processing and left holes in the material. Staining with Safranin-O was performed to further distinguish the alginate (red) from the gelatin (green) and confirm distribution of the GMPs throughout the scaffold (Figure 1D). The alginate contains regional density differences, presumably caused by uneven degree of crosslinking. The addition of BCP to the constructs did not influence scaffold integrity and the strands were successfully printed and remained intact, shown by HE staining (Figure 1E).

### Osteogenic differentiation in vitro

**Figure 3:**
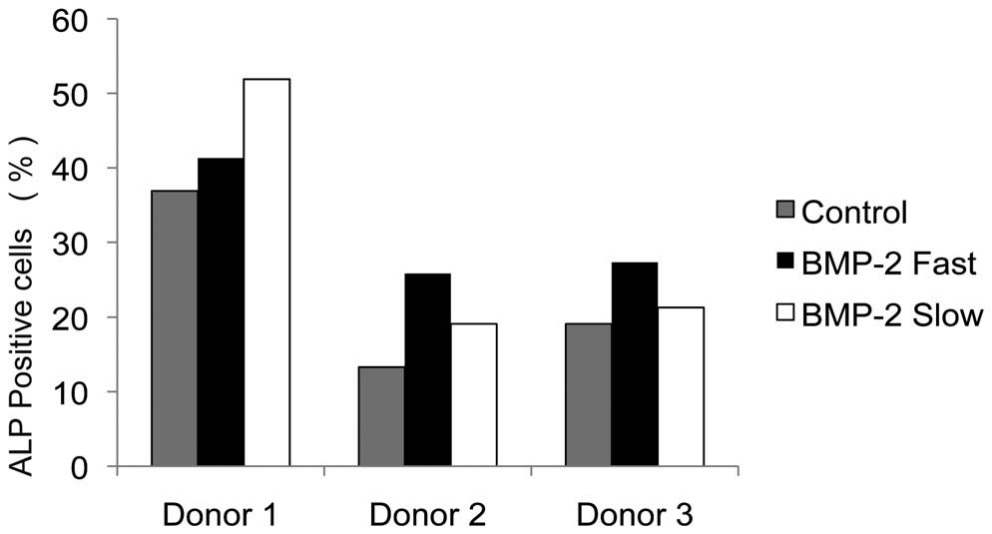
Bioactivity of released BMP-2. Frequency of ALP positive MSCs after culturing for three weeks without BMP-2 (control), with BMP-2 in the hydrogel (fast release), and with BMP-2 laden GMPs (slow release), determined for 3 individual donors. Statistical analysis performed on mean values per donor.

**Figure 4:**
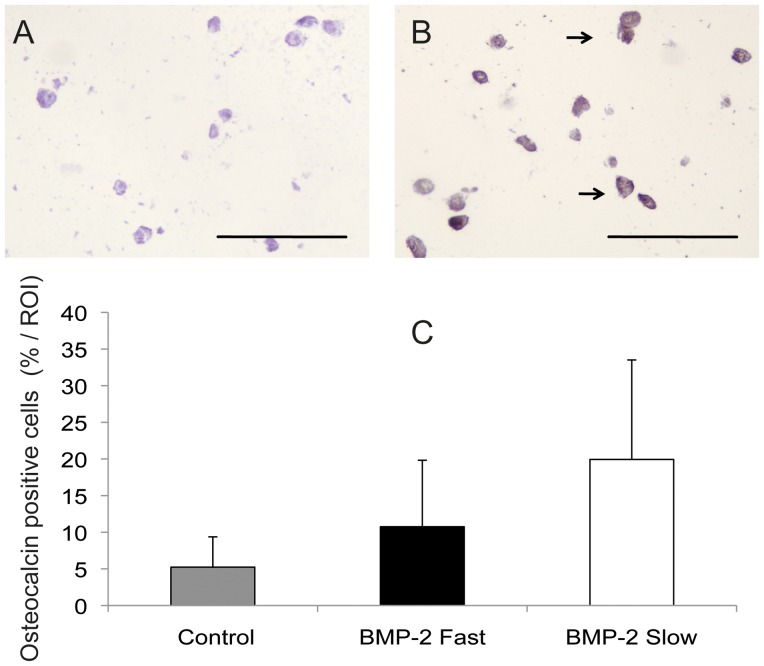
Osteocalcin staining of bioprinted scaffolds after 3 weeks of culturing. A. Control staining (isotype-matched control antibody); B. Osteocalcin positive cells from a scaffold with slow release of BMP-2. Arrows indicate some of the positively stained cells (brown). Scale bars represent 50 µm; C. Percentage of osteocalcin positive cells per region of interest, no statistically significant differences were found. Results are presented as mean±SD.

The constructs were analyzed for osteogenic differentiation after three weeks of culturing in expansion medium, by both detection of ALP expression using FACS and immunohistochemistry for osteocalcin. After dissolving the scaffold in citrate buffer flow cytometry was performed. The number of ALP positive cells was determined for all three conditions, as shown in Figure 3. Low signal of the isotype matched control antibody group confirms specific binding of the antibody. In one of the donors the number of ALP positive cells increased when BMP-2 was present for a longer period of time, in two donors the number of ALP positive cells decreased. A beneficial effect of prolonged protein presence was not seen, as the differences between the experimental groups are not statistically significant. In the immunohistochemical staining for osteocalcin the isotype-matched control staining was negative (Figure 4A). In the control constructs 5.2±4.1 % of the cells stained osteocalcin positive. In the fast release group 10.7±9.1% of all cells were expressing osteocalcin. In the slow release group osteogenic differentiation was seen in 19.9±13.6 % of the cells (Figure 4B). There was no significant difference between the groups (p = 0.2). Arrows indicate positively stained cells.

### Osteogenic differentiation in bioprinted scaffolds

**Figure 5:**
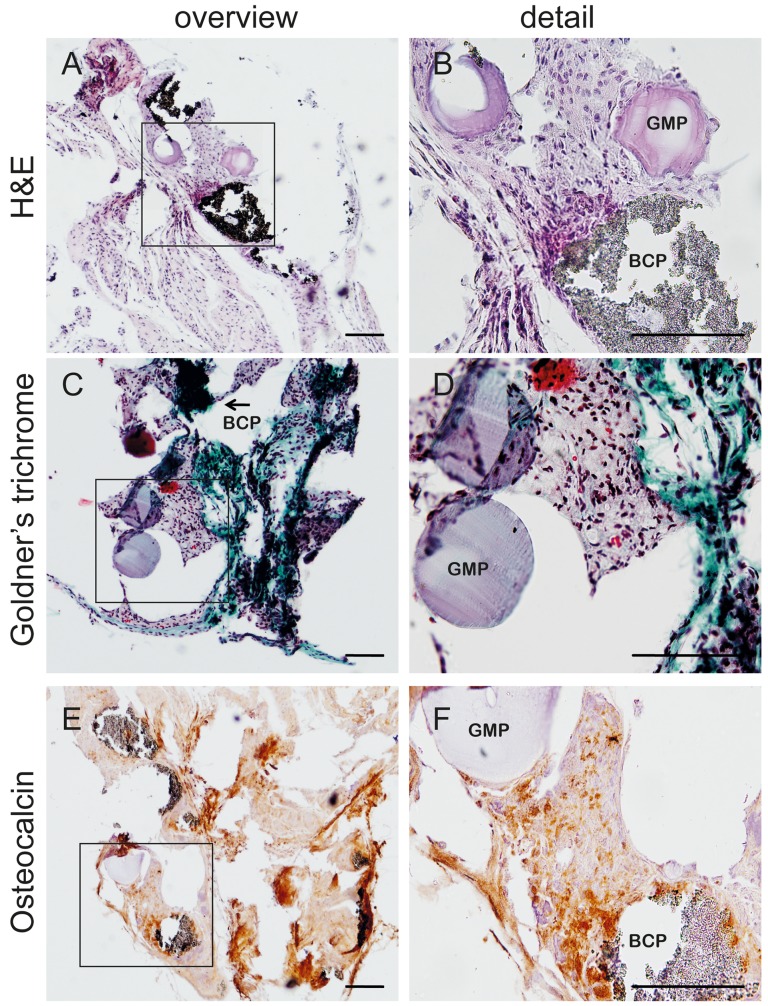
Osteogenic differentiation *in vivo*. Presence of gelatin microparticles (GMP) and biphasic calcium phosphate granules (BCP) after 6 weeks of subcutaneous implantation in mice as indicated in the pictures. An overview and detail of HE, Goldner's trichrome and osteocalcin stainings are shown. Scale bars represent 100 µm.

After 6 weeks of in vivo implantation in mice the scaffolds were explanted and examined using immunohistochemistry. The alginate scaffolds were degraded to such an extent that scaffold retrieval was challenging, some explants were too small to analyze for osteogenic differentiation. Larger pieces of retrieved scaffolds (all from the fast release group) were suitable for histological analysis (Figure 5).

In the retrieved scaffolds GMPs and BCP particles were clearly visible on the HE staining. In the Goldner's trichrome staining collagen depositions (in green) were observed. Cells positive for osteocalcin were present in the regions around both BCP and GMPs, which indicates osteogenic differentiation at these locations. No alginate was present in these tissues, as indicated by staining with Safranin-O (data not shown). This indicates that the loss of scaffold integrity occurred rapidly. To further investigate this fast alginate degradation, two alginate/BCP scaffolds were implanted subcutaneously in mice, and already after one week alginate degradation and cell invasion was evident (data not shown).

### Bone formation in porous BCP cylinders

**Figure 6:**
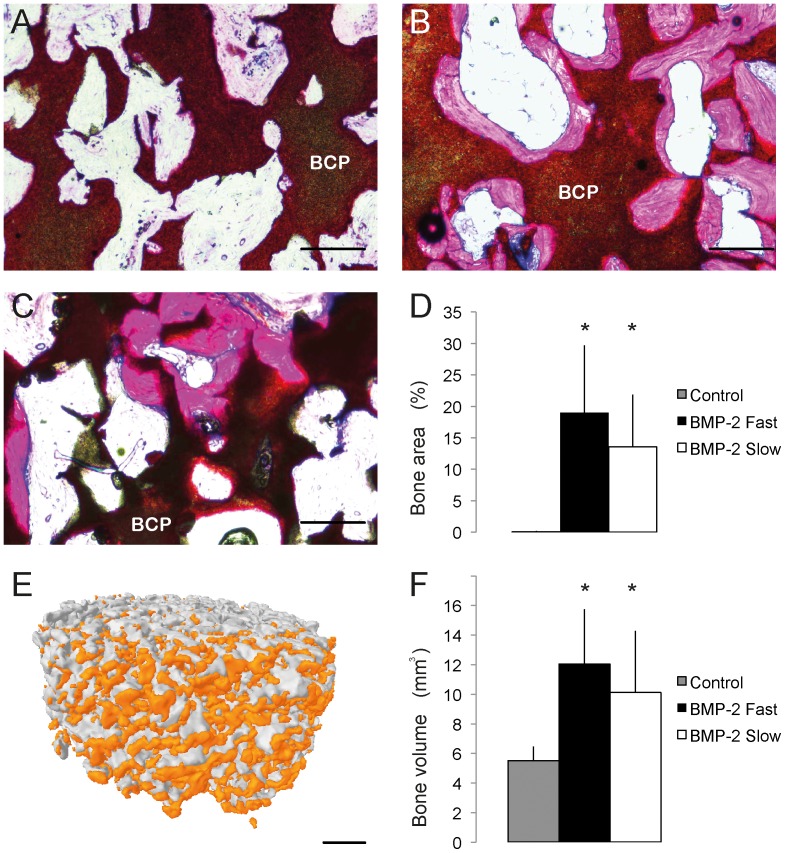
Bone formation after 12 weeks of subcutaneous implantation in rats. A. Control group; B. Fast release of BMP-2 group; C. Sustained BMP-2 released from GMP group. Section were stained with methylene blue and basic fuchsin. Biphasic calcium phosphate (BCP) is indicated in the pictures. D. Quantification of bone content by histomorphometry, n = 10. E. 3D reconstruction of micro-CT showing bone formation (orange) in a scaffold of the BMP-2 fast group. F. Quantification of bone content by micro-CT, n = 10. Representative pictures from each group are given. Scale bars represent 100 µm in A-C, 1 mm in E; Results are presented as mean ± SD, * p<0.01 compared to control.

To analyze whether BMP-2 release kinetics influenced the extent of bone formation, a second animal study was performed in rats. Porous BCP cylinders (control, fast or slow release) were implanted subcutaneously to ensure scaffold integrity throughout the experiment. Bone formation in BCP cylinders was analyzed using histomorphometry and micro-CT after 12 weeks of subcutaneous implantation (Figure 6). The control group did not form bone (0±0 %) in the BCP cylinders, which was significantly lower than the BMP-2 containing groups (p<0.01, in both histomorphometry and micro-CT). A bone volume of 5.5±0.9 mm^3^ was measured in this group with micro-CT. In the slow release group a bone area of 13.5±8.3 % from the histological sections and bone volume of 10.1±4.2 mm^3^ from the CT data were measured. In the fast release group the average bone area% reached 19±10.7 %, and the bone volume 12.1±3.7 mm^3^.

## Discussion

This study has shown that prolonged presence of BMP-2 in scaffolds can be accomplished by applying controlled delivery microparticles, such as GMPs. It is feasible to incorporate these GMPs into a bioprinted alginate construct containing BCP and gMSCs. The presence of BMP-2 in bioprinted hydrogel constructs has led to osteogenic differentiation *in vitro* and *in vivo*. The ability to place and release growth factors in such a way is novel and offers many possibilities in terms of scaffold architecture and bioactivity.

The 3% pre-crosslinked alginate was printable and could be rapidly crosslinked after printing, but *in vivo* it was degraded very fast, which severely affected scaffold integrity. The hydrogel with incorporated BMP-2, BCP granules and gMSCs probably dispersed through the pocket. The lack of coherence likely explains the absence of bone formation after in vivo implantation, as each of the components has been successfully used in bone tissue engineering approaches in previous experiments by our group [31], [32]. The porosity of the constructs may have added to the disintegration effect, as it enlarges the surface to volume ratio of the constructs [33]. It is known that ionically cross-linked alginate loses mechanical properties over time due to an outward flux of ions into surrounding culture medium [34], [35]. In the future, other measures need to be taken to be able to make low percentages of alginate printable, or combinations of hydrogels should be used as scaffold material to retain structure until bone starts to form.

Many different biomaterials are suitable for controlled release, gelatin being well-known for its prolonged growth factor delivery, characterized by a small initial burst and showing no tissue damaging residual material. As such, gelatin is used in many FDA-approved devices. The release profile of BMP-2 shown here has two distinct phases, starting with a burst release of about 30% growth factor. It is known from literature on gelatin release kinetics that this burst is due to diffusion from the outer regions of the particles [36]. The second phase, which shows sustained release is suggested to be based on enzymatic degradation of the gelatin, thereby releasing the bound BMP-2. When collagenase was not present in the release medium this second phase hardly occurred. Detection of the released protein was measured using radioactive labeling as well as ELISA, both widely used assays to assess BMP-2 concentrations. Still, these highly quantitative methods do not take into account the loss of protein due to breakdown and surface adhesion.

The *in vitro* release data shown in this article are probably not in accordance with *in vivo* release rates, which can be significantly faster due to higher enzymatic activities. By using collagenase in the experiments, the *in vivo* degradation was at least partly mimicked, but we expect *in vivo* release to be faster still. Also, a 100% release was not reached after 4 weeks, indicating there was still residual growth factor present in the experimental samples. Give the observation that *in vitro* and *in vivo* release often show different profiles [22], we can only estimate the total dose which has become available in the implanted constructs. Dosage of BMP-2 in the *in vitro* experiments appeared to be sufficient, as prolonged presence of BMP-2 released from GMPs has led to osteogenic differentiation of the cells.

In this study we used goat MSCs as a model system, because this large animal model is widely used in preclinical studies aiming at translation to the clinic and thus in generating cm-scaled bone constructs. The presence of porosity in larger constructs promoted tissue ingrowth and vascularization, which was demonstrated before using the bioprinting technology [37]. When applying the GMPs in bioprinted scaffolds, it appeared that inclusion of BMP-2 through these formulations containing growth factors is feasible. Bone formation by application of BMP-2 laden GMPs was studied, and results were analyzed using both histomorphometry and micro-CT. Although micro-CT analysis poses advantages such as rapid analysis of entire constructs and *in vivo* analysis, there is a discrepancy with histomorphometry measurements, as is illustrated by the control group. We know from histomorphometry that no bone was present in these constructs, yet a considerable signal was detected with micro-CT. This is presumably caused by border-artefacts from the BCP, that has a radiolucency close to to bone. Despite this over-estimation, the relative increase in bone formation between the groups is comparable, which corresponds to literature reporting good correlations between micro-CT and histomorphometry [38]. Histomorphometry however remains the gold standard to analyze bone formation. Growth factor induced bone formation was analyzed, and growth factor presence either by slow or fast release led to a significantly higher bone area % compared to growth factor free formulations in the rat ectopic model. This is in not in accordance with earlier studies in for instance dogs, where the faster BMP-2 release led to higher ectopic bone volume [39]. Apart from location specific differences, also species differences play a role, making clinical translation even more complicated.

## Conclusion

We conclude from the current investigation that prolonged BMP-2 release can be accomplished by loading the growth factor on GMPs. The BMP-2 released from the GMPs in these scaffolds is biologically active. Presence of BMP-2 in bioprinted hydrogel constructs has led to osteogenic differentiation, from which we conclude that it is feasible to bioprint a controlled release system. BMP-2 significantly increased bone formation, the release timing did not influence the bone volume within the constructs.

The ability to place and release growth factors in 3D printed constructs is novel and offers many possibilities in terms of scaffold architecture and bioactivity.
